# Spinal Surgery

**Published:** 1894-05-26

**Authors:** 


### PROGRESS IN SURGERY.

### SPINAL SURGERY.

Interest continues to centre greatly in the operation of trephining the spine in cases of damage to the cord from fracture, blood extravasation, spondylitis, and tumour. Recently, valuable additions—several of which are referred to below—have been made to the records on this subject, but the difficulty of diagnosis in this class of case prevents surgeons from formulating in any definite way the indications for operating.

*Fractures and Dislocations.*—From clinical and experimental observations in connection with fractures in the cervical region Dr. Manly[1] draws, amongst others, the following conclusions: (1) Grave injuries of the spinal cord, from injury in the neck, without fracture, are rare; (2) when paralysis immediately follows cervical injury it clearly establishes the fact that the cord has borne serious damage; (3) fractures which involve the respiratory centres (from the atlas to the fifth) are almost inevitably mortal; (4) those below this point are sometimes within the range of operative relief when the apophyses alone are involved, and are not so dangerous to life.

Dr. Detweiler[2] records a case of dislocation in the lumbar region with complete paralysis of the parts below, in which reduction was obtained. The patient —a miner—was given ether, and the body having been fixed to the head of the bed, strong extension was made on the lower limbs, judicious pressure being at the same time applied to the spine and abdomen. The dislocated parts returned to their places with a loud snap. The patient ultimately made a good recovery. Walton[3] describes a method for reducing dislocation in the cervical region which he has found successful.

*Pott's Disease.*—Binaud and Crozet[4] state that the best results from trephining for spinal caries have been met with in instances of paraplegia due to sclerous perimeningitis, to fungous compression of the medulla, and to intraspinal collections of pus. On the other hand, extensive osseous lesions and intrameningeal tuberculous infection are unfavourable conditions for operation. They record two cases operated on by Pitres for the relief of paraplegia due to Pott's disease. Both patients were adults. In one case sensibility was seriously impaired. At the operation a large quantity of pus was evacuated from the spinal canal, and the dura mater found thickened and fungoid. No improvement followed, and death took place two months later from pulmonary tuberculosis. In the other case, sensibility was only slightly impaired. Extradural masses of tuberculous deposit were found and scraped away. Gradual return of the functions of the lower limbs took place, and at the end of a year the patient was able to move about on crutches. Dr. Barton,[5] in dealing with the treatment of Pott's disease in the early stage, before there is marked angular deformity, insists that any support to be efficient must maintain the spine in a position in which the deformity is removed. He contends that suspension during the application of a plaster jacket does not in any essential degree modify the deformity. He advocates the following method: A plaster of Paris jacket is applied to the patient and removed when set. It is then filled with plaster of Paris, and a solid cast is thus obtained of patient's figure. From this cast the deformity is removed by modelling so as to make the cast correspond with the patient's normal figure. To this cast the jacket—preferably of perforated leather—is accurately fitted and then applied to the patient.

*Tumours.*—In 1887 Victor Horsley, by the successful extirpation of a tumour of the cord, opened up a new field in the treatment of cases formerly regarded as hopeless. Similar operations have since been performed by others with varying success. Recently, Dr. Ransom[6] gave a detailed report of a case with symptoms of four months' duration, in which he diagnosed tumour in the dorsal region probably extradural. Mr. Joseph Thompson resected the spine, and an extradural tumour, which proved to be a sarcoma, was removed. The patient sank and died two days after operation. Caselli[7] records the successful removal of an osteoma from the spinal canal of a woman aged 28, suffering from pressure symptoms of seven months' duration. A case of hydatid cysts in the vertebral canal is reported by Friedeberg.[8] The symptoms were gradual in onset, and were present for several years. For some time previous to death motor and sensory paralysis of the lower limbs, paralysis of rectum and bladder, trophic changes, loss of electrical reactions and the reflexes, were present. At the autopsy small hydatid cysts were found in the disorganised sacrum, and there were also cysts here and there outside the dura mater, as high as the second dorsal vertebra. H. G. Turney and H. H. Clutton[9] record a case in which the spine was resected, and a myxomatous growth removed. Distinct improvements followed as regards sensibility and movement in the lower limbs, but patient died ten days after operation from sepsis.

*Spina Bifida.*—Treatment by the open method is gaining ground, and many surgeons now prefer to treat spina bifida by incision and excision of the sac rather than by injection with Morton's fluid. Some go farther still, and attempt to close the bony cleft by means of an osteoplastic operation. Dr. Roberts[10] reports a case of excision of the sac: hæmorrhage and sepsis caused the death of the child fifteen days after operation. Notwithstanding the fatal issue in this case, he advocates the open method of treatment. Dr. Herricks[11] has successfully excised the sac in a child six weeks old.

[1] Med. Record, Oct. 28, 1893. [2] Therapeut. Gaz., 1893, p. 800. [3] Amer. Journ. Med. Sc., Dec., 1893, p. 735. [4] Brit. Med. Journ., Feb. 17, 1894, Ep. p. 25. [5] Intern. Med. Mag., Nov., 1893. [6] Brit. Med. Journ., Feb. 24, 1894, p. 395. [7] Brit Med. Journ., Dec. 23, 1893. Ep. p. 102. [8] Brit. Med. Journ., Mar. 3, 1894. Ep. p. 33. [9] Lancet, Feb. 17, 1894, p. 398. [10] Annals of Surgery, March, 1894. [11] New York Med. Journ., Dec. 30, 1893.

